# Meteorological Table

**Published:** 1812-10

**Authors:** 


### METEOROLOGICAL TABLE.

### *From the 26th of August, to the 25th of September.*

| | D. | Therm. | | | Barom. | | Hygrom. Dry. | | | Damp. | | | Winds. | Atmos. Variation. |
|---|---|---|---|---|---|---|---|---|---|---|---|---|---|---|
| | 27 | 62 | 71 | 58 | $29^9$ | 30 | 9 | 22 | 14 | | | | N.. | F.. —.... |
| | 28 | 55 | 60 | 55 | | | 10 | 12 | 10 | | | | NE. | F. C.. |
| ◑ | 29 | 55 | 57 | 55 | | | 4 | — | — | — | 1 | 3 | N. | R.. C.. R. |
| | 30 | 56 | 61 | 58 | | | — | 3 | 5 | 5 | — | — | N. | R. C.. F. |
| | 31 | 58 | 63 | 57 | $30^1$ | — | — | 5 | 3 | 1 | — | — | N. | C.. |
| | 1 | 59 | 63 | 56 | $30^2$ | — | 3 | 10 | 4 | | | | NE. | C. |
| | 2 | 57 | 64 | 60 | — | $30^1$ | 2 | 10 | 3 | | | | NE.. | C.. |
| | 3 | 59 | 65 | 61 | | | 2 | 10 | 3 | | | | N. | C.. |
| | 4 | 61 | 64 | 60 | $29^9$ | — | 1 | 4 | 3 | | | | N. | F.. |
| ● | 5 | 58 | 61 | 60 | | | 1 | 12 | 7 | | | | E.. | F. |
| | 6 | 60 | 59 | 63 | — | 30 | — | 19 | 10 | 1 | — | — | E. | F.. |
| | 7 | 57 | 70 | 59 | $30^1$ | — | 6 | 10 | 12 | | | | E. | C.. F... |
| | 8 | 53 | 71 | 62 | — | 30 | 7 | 15 | 10 | | | | S. | C.. F... |
| | 9 | 63 | 67 | 58 | | | 4 | 1 | 4 | | | | W.. | C.. F.. C. R.. in N |
| | 10 | 59 | 68 | 60 | — | $30^1$ | 1 | 10 | 14 | | | | W. | R. F.. |
| | 11 | 56 | 70 | 60 | $30^2$ | $30^3$ | 6 | 12 | 16 | | | | NW.. | F.. —... |
| | 12 | 56 | 70 | 62 | | | 7 | 17 | 12 | | | | W. | C. F... |
| ◐ | 13 | 57 | 72 | 62 | — | $30^2$ | 8 | 24 | 15 | | | | SW.. | F... |
| | 14 | 54 | 71 | 62 | | | 10 | 21 | 16 | | | | W. | C.. F.... |
| | 15 | 57 | 70 | 65 | $30^1$ | — | 10 | 24 | 13 | | | | W. | F.. —.... |
| | 16 | 58 | 74 | 61 | 30 | $29^9$ | 9 | 32 | 14 | | | | W. | F... |
| | 17 | 57 | 60 | 53 | | | 9 | 6 | 5 | | | | NW. | C. R.. F.. |
| | 18 | 51 | 59 | 50 | 30 | $30^2$ | 6 | 12 | 16 | | | | NW.. | F. |
| | 19 | 48 | 67 | 57 | | | 9 | 23 | 15 | | | | W. | C.. F.. |
| ○ | 20 | 59 | 69 | 58 | — | $30^1$ | 10 | 28 | 25 | | | | NW.. | F. —... |
| | 21 | 68 | 73 | 60 | 30 | — | 15 | 26 | 18 | | | | SW.. | F.. |
| | 22 | 57 | 68 | 58 | | | 11 | 3 | 2 | | | | NW. | R. —... —.. in N |
| | 23 | 58 | 58 | 53 | | | 3 | 5 | 6 | | | | N. | C. |
| | 24 | 53 | 56 | 53 | | | 5 | 10 | 14 | | | | N. | C.. |
| | 25 | 50 | 61 | 56 | $30^1$ | — | 14 | 18 | 11 | | | | S.. | C... |

Quantity of rain $\frac{52}{100}$ of an inch. The quantity of rain in this interval has been very little more than half an inch, constituting by far the dryest month in the year. The generally pleasant and dry month of September has presented a striking contrast to August, which was cold, stormy, and wet. We are assured that on the night of the 15th of August, some of the Kentish hills were covered with *snow*.

Some cases of cholera have occurred, connected, as it appeared, with the increased heat of the weather. Tertian intermittents, in high and dry situations near London, have been met with; and inflammatory affections of the lungs in young children have also appeared, of a severe and dangerous character. Blood drawn from the chest by leeches, and especially the great, and often to the parents alarming, flow that succeeds their application, was decidedly beneficial.

